# Can Experienced Observers Differentiate between Lipoma and Well-Differentiated Liposarcoma Using Only MRI?

**DOI:** 10.1155/2013/982784

**Published:** 2013-12-09

**Authors:** Patrick W. O'Donnell, Anthony M. Griffin, William C. Eward, Amir Sternheim, Lawrence M. White, Jay S. Wunder, Peter C. Ferguson

**Affiliations:** ^1^Department of Orthopaedic Surgery, Markey Cancer Center, University of Kentucky, 740 S. Limestone Avenue, Lexington, KY 40536, USA; ^2^Department of Orthopaedic Surgery, Mount Sinai Hospital, Toronto, ON, Canada M5G 1X5; ^3^Department of Orthopaedic Surgery, Duke Cancer Center, Duke University, Durham, NC 27710, USA

## Abstract

Well-differentiated liposarcoma represents a radiographic diagnostic dilemma. To determine the accuracy, interrater reliability, and relationship of stranding, nodularity, and size in the MRI differentiation of lipoma and well-differentiated liposarcoma, MRI scans of 60 patients with large (>5 cm), deep, pathologically proven lipomas or well-differentiated liposarcomas were examined by 10 observers with subspecialty training blinded to diagnosis. Observers indicated whether the amount of stranding, nodularity, and size of each tumor suggested a benign or malignant diagnosis and rendered a diagnosis of lipoma or well-differentiated liposarcoma. The accuracy, reliability, and relationship of stranding, nodularity, and size to diagnosis were calculated for all samples. 69% of reader MRI diagnoses agreed with final pathology diagnosis (95% CI 65–73%). Readers tended to err choosing a diagnosis of liposarcoma, correctly identifying lipomas in 63% of cases (95% CI 58–69%) and liposarcomas in 75% of cases (95% CI 69–80%). Assessment of the relationship of stranding, nodularity, and size to correct diagnosis showed that the presence of each was associated with a decreased likelihood of a lipoma pathological diagnosis (*P* < 0.01). While the radiographic diagnosis of lipoma or well-differentiated liposarcoma cannot be made with 100% certainty, experienced observers have a 69% chance of rendering a correct diagnosis.

## 1. Introduction

Well-differentiated liposarcoma presents a diagnostic dilemma to the treatment team due to the inaccuracy of biopsy and the difficulty in differentiating these low-grade tumors from benign lipomas on imaging alone [1–4]. This difficulty in diagnosis can lead to patient worry, delays in diagnosis, and a considerable risk for local recurrence with inappropriate surgical resection. As well-differentiated liposarcomas show low to no metastatic potential, with appropriate excision, low rates of recurrence can be expected [5–8].

**Figure 1:**
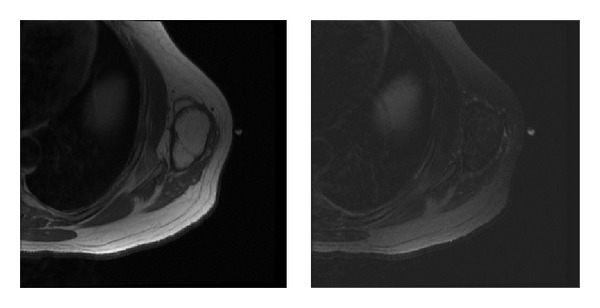
Axial T1 and T2 MRIs of a patient with a fatty tumor. Despite the benign appearance of this tumor radiographically, final pathological review confirmed a diagnosis of low-grade liposarcoma.

**Figure 2:**
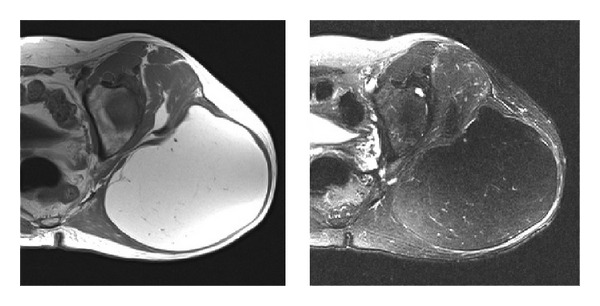
Axial T1 and T2 MRIs of a patient with a large fatty tumor in the buttock. Despite the large size and intratumoral stranding/nodularity, final pathological review confirmed a diagnosis of lipoma.

MRI serves as an excellent modality to discern certain soft tissue tumors like lipomas given their specific imaging characteristics [1, 3]. Low-grade fatty tumors represent a radiographic spectrum from innocent appearing homogeneous lesions, isointense to subcutaneous adipose tissue on all sequences to the more ominous appearing lesions with heterogeneity (Figures 1 and 2). The ideal treatment situation for these low-grade fatty tumors would be one where the pathological diagnosis would correspond perfectly with the imaging characteristics. However, this pathological to radiological correlation does not occur in the majority of cases (Figures 1 and 2) [2, 5, 9]. As such, musculoskeletal radiologists and orthopaedic oncologists have evolved to include well-differentiated liposarcoma on the radiographic differential of suspicious fatty lesions.

Several authors have evaluated the sensitivity and specificity of specific MRI sequences in the diagnosis of well-differentiate liposarcoma; however, the diagnostic reliability between specialties (musculoskeletal radiology and orthopaedic oncology) has not been well characterized [1–4, 10]. In addition, specific variables that may portend an elevated likelihood of well-differentiated liposarcoma (stranding, nodularity, and size) have been discussed in the literature extensively, without agreement among the experts [1–4, 10].

The purpose of this study was therefore to evaluate the accuracy, reliability, and association between the variables of size, stranding, and nodularity in the diagnosis of lipoma versus well-differentiated liposarcoma based on MRI imaging alone. In addition, the accuracy and reliability of trained musculoskeletal radiologists and orthopaedic oncologists in the radiographic diagnosis of these tumors were assessed to see if any differences existed between specialties. Our hypothesis was that experienced readers in both specialties blinded to diagnosis would be unable to differentiate between a series of fatty lesions and that the variables of size, nodularity, and stranding would show no predictive association with these diagnoses. If true, this study would support the argument to treat all low-grade fatty lesions with marginal excision regardless of the MRI interpretation or concern regarding variables of size, nodularity, or stranding.

## 2. Materials and Methods

After research ethics board approval, MRI scans of 60 patients with large (>5 cm in greatest dimension), deep, pathologically proven lipomas (31 patients) or well-differentiated liposarcomas (29 patients) were identified from a prospectively collected database. Pathological review and diagnosis were made by one of three pathologists with subspeciality training in musculoskeletal oncology and sarcoma pathology following WHO criteria for the classification of sarcoma. Informed consent was obtained from all research subjects as a part of their enrolment into this database. Patient MRI scans were retrospectively examined by 10 observers with subspecialty training in musculoskeletal radiology or orthopaedic oncology, blinded to diagnosis. There were 2 attending staff physicians and 3 fellows from each orthopaedic oncology and musculoskeletal radiology who participated.

**Figure 3:**
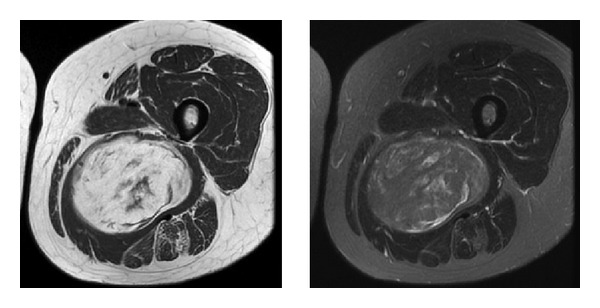
Axial T1 and T2 MRIs showing intralesional stranding in a fatty tumor. Final pathological review confirmed a diagnosis of low-grade liposarcoma.

**Figure 4:**
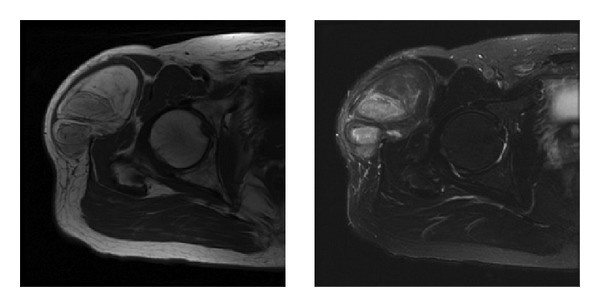
Axial T1 and T2 MRIs showing intralesional nodularity in a fatty tumor. Final pathological review confirmed a diagnosis of low-grade liposarcoma.

After reviewing each patient MRI on a PACS viewing module, observers indicated whether the amount of stranding, nodularity, and size of each tumor was concerning for well-differentiated liposarcoma and rendered a diagnosis of lipoma or well-differentiated liposarcoma. To simulate a clinical scenario, each reader was given the ability to use whatever image sequences available for diagnostic purposes (see later). Stranding (Figure 3) was defined as linear tissue heterogeneity present within the mass, and nodularity (Figure 4) was defined as intralesional tissue heterogeneity that was not linear. The concerning size variable was left to the individual readers discretion for interpretation; however, all tumors were greater than 5 cm in greatest dimension as mentioned above. All variables were categorical and recorded as either “yes” or “no” for the measurements of concerning size, nodularity, and stranding and whether the reader was concerned about a diagnosis of well-differentiated liposarcoma, based on these individual variables. For example, while all lesions were >5 cm, each reader had to independently determine whether the size of the lesion, amount of stranding, or nodularity were concerning for a liposarcoma diagnosis. Diagnosis categorical variables of “lipoma” or “well-differentiated liposarcoma” were also recorded for each tumor.

As the variables of size, stranding, and nodularity are used clinically by radiologists without standardization criteria to interpret such images and render a diagnosis, these variables were intentionally not standardized for individual readers for the purpose of this study. This was done to simulate a clinical scenario and therefore better evaluate the ability of readers to render a correct diagnosis and to determine the reliability and validity of reader diagnoses towards these tumors.

All imaging was performed with 1.5-T MRI system (Signa, GE Medical System, Milwaukee, WI) using a surface coil or body coil depending on the anatomic location. Specific imaging protocols included at least two planes of T1-weighted images (TR 500 ms/TE 10 ms) and fast spin echo T2-weighted images (TR 4800 ms/TE 100 ms) with selective fat saturation. STIR sequences (inversion time 110–150 msec) were also occasionally available. In all of the cases reviewed, either T2W fast spin echo imaging with spectral fat suppression or STIR imaging was performed. Imaging sequences used by readers of this study for diagnostic purposes were performed without contrast agents.

Kappa statistics along with 95% confidence limits were used to determine the reliability of each categorical measurement [11]. The interobserver reliability was evaluated by determining the agreement among the observers. *P* values for comparison to “no agreement” were generated from an exact test [12]. All *P* values were two-sided and any *P*-values less than 0.05 were considered statistically significant. Sixty MRI studies resulted in 95% power of 0.8 based on a type I error of 5%. Data analysis was performed using SAS version 9.2 computer software.

## 3. Results

### 3.1. Accuracy

413/600 (69%) reader diagnoses agreed with the final pathological diagnosis. The 95% confidence limits for the accuracy percentage were (65%–73%). If the pathology diagnosis was lipoma, readers correctly identified lipoma in 196/310 (63%) cases (95% CI 58%–69%). If the pathology diagnosis was well-differentiated liposarcoma, readers correctly identified liposarcoma in 217/290 (75%) cases (95% CI 69%–80%). All readers chose liposarcoma more frequently than lipoma. There was no difference regarding diagnostic accuracy when compared between the specialties of orthopaedic oncology and musculoskeletal radiology (average 68%, 95% CI 63%–74% versus 69%, 95% CI 64%–75%, resp.). Attending physicians had a slightly high rate of diagnostic accuracy when compared to fellows (average 71%, 95% CI 70%–72% versus 67%, 95% CI 61%–73%, resp.).

### 3.2. Reliability

Table 1 shows the interrater reliability for diagnosis and each of the categorical variables of stranding, nodularity, and size. Interrater reliability for diagnosis showed “substantial” agreement with a kappa estimate of 0.63 (95% CI 0.61–0.65). Interrater reliability showed only “slight” agreement for stranding and size but “moderate” agreement for nodularity (kappa estimates 0.17, 0.11, and 0.41; (95% CI 0.14–0.19, 0.09–0.13, 0.39–0.43, resp.).

### 3.3. Relationship of Stranding, Nodularity, and Size to Diagnosis

Tables 2 and 3 show the relationships of stranding, nodularity, and size to the diagnosis of liposarcoma. Positive reader responses for stranding and nodularity were associated with an increased likelihood of liposarcoma diagnosis (*P* < 0.01). Although this combination had a higher probability of a true liposarcoma diagnosis, readers overdiagnosed liposarcoma by a large margin in this instance. The inverse is true if readers choose “no” for both stranding and nodularity, which was associated with a lipoma diagnosis. A positive response for size was also associated with increased likelihood of a liposarcoma diagnosis (*P* < 0.01), although the misdiagnosis gap was not as large as for stranding/nodularity, as readers were overall more likely to choose a diagnosis of liposarcoma. There was no difference regarding interpretation of these categorical variables or their relationship towards the ultimate diagnosis when compared between specialties.

## 4. Discussion

Well-differentiated liposarcomas are low-grade fatty tumors with negligible metastatic potential and less than 10% chance of local recurrence even after positive margin excision [7, 8, 13]. Well-differentiated liposarcomas do however represent a diagnostic dilemma given the difficulty discerning well-differentiated liposarcoma from lipoma on imaging and problems associated with sampling error during biopsy [2]. Due to poor understanding of the biology of well-differentiated liposarcomas or radiologic phrases like “liposarcoma cannot be ruled out, referral to orthopaedic oncologist is recommended,” these tumors are the cause of unnecessary referral to tertiary sarcoma centers and more importantly patient worry. As such, a standardized approach to the radiographic evaluation and treatment of these lesions would be beneficial to both patients and the physicians who care for them.

The goal of this study was to determine whether experienced observers in musculoskeletal radiology and orthopaedic oncology could differentiate between lipomas and well-differentiated liposarcomas on imaging alone and to determine the relative association of variables such as stranding, nodularity, and size to this diagnosis. Our hypothesis was that even experienced observers would be unable to differentiate between lipomas and well-differentiated liposarcomas. Therefore, if treating physicians were unable to accurately diagnose these tumors on imaging or via biopsy, an argument suggesting that all symptomatic low-grade fatty lesions may be treated with marginal excision could be supported. While the results presented here are better than chance alone, they do reaffirm that a definitive diagnosis cannot be made on imaging alone until better imaging criteria are identified.

We also present data here that supports several other variables surrounding the radiographic diagnosis of low-grade fatty lesions. First, there is a substantial interrater reliability based on kappa statistics towards making the radiographic diagnosis. These data are based on a high number of readers and the categorical variables of “lipoma” versus “liposarcoma.” In addition, the relative accuracy of observers from musculoskeletal radiology and orthopaedic oncology with various levels of training (fellow versus staff) was not statistically different. Finally, the variables of size, nodularity, and stranding did show an association with the diagnosis of well-differentiated liposarcoma and as such based on these data should continue to be used in the radiographic interpretation of these tumors.

Other authors have evaluated the utility of MRI in the differentiation of well-differentiated liposarcoma from lipoma and determined the value of fluid-sensitive imaging sequences in making this distinction [2, 3, 10]. Doyle et al. evaluated the observer performance using T1-weighted spin echo and fluid-sensitive MRI sequences [1]. They found sensitivity over 94% for T1-weighted images and 100% for fluid-sensitive sequences. Specificities however for these same imaging sequences were 76% and 64% for the T1-weighted sequences and 70% and 73% for the fluid-sensitive images. The interobserver agreement was rated as “good” based on kappa statistics. This study however only had two observers, both radiologists, and a small representative number of well-differentiated liposarcomas in the study design (18 of 51 tumors). In addition, these authors attempted to correlate the radiographic findings with pathological variables like fibrosis, stranding, and nodularity; however, they were unable to make associations between these pathological variables and the observed radiographic parameters. As such, Doyle et al. question the diagnostic importance of nodularity, stranding, and size in the differentiation of lipomas from well-differentiated liposarcomas on imaging alone. Our data however suggests that the variables of nodularity, stranding, and size do show association with the correct radiographic diagnosis and therefore should continue to be used despite the poor correlation to pathological variables. One key point in the differentiation between the presented data and that of Doyle et al. is that the histological variable of fibrosis may not correlate with the radiographic viable of stranding. As those authors did not specifically map this fibrosis within the pathological specimen to areas specified on the MRI, we question the validity of the author's conclusions.

The accuracy of diagnosis presented here (69%) is lower than that documented in previous reports [1–3]. In one such report, Gaskin and Helms retrospectively reviewed 126 lesions and determined the sensitivity, specificity, and accuracy of MRI in making the distinction between lipoma, lipoma variants, and well-differentiated liposarcoma [10]. They reported an overall accuracy in their study of 83%; however, there are several differences between that study and data presented here. Gaskin and Helms selected cases through a radiological database search by identification of key phrases (such as “lipo-” or “fat”) in the radiological report. Only after selection of 126 cases which other radiologists had been confident enough to use such terms were the pathological diagnoses reviewed. This method of case inclusion represents a selection bias given the prior documentation by reading radiologists of the terms “lipo-” or “fat.” Lesions with concerning heterogeneity, nodularity, or stranding in which the MRI evaluation suggested a more aggressive diagnosis than a lipomatous or fatty tumor would be excluded using this method of selection. Given the heterogeneity of fatty tumors and the fact that the pathological diagnosis is the most valid, our presented accuracy may be appropriate. The second difference between the study by Gaskin and Helms was that only 64 of 126 tumors were resected at their tertiary institution and available for review by their trained musculoskeletal pathologist. Again, given the heterogeneity within the diagnosis of well-differentiated liposarcoma and the specific diagnostic criteria surrounding this diagnosis, the presented radiological accuracy in that study may not be truly representative of all low-grade fatty lesions [9].

Data presented here suggests that the variables of size, nodularity, and stranding show an association with the ultimate pathological diagnosis and as such should continue to be used in the radiographic interpretation [2]. It has been our experience that size is the variable most often used by referral centers or radiologists to suggest a diagnosis of well-differentiated liposarcoma but the variable least used by our treatment team in making the ultimate decision a MRI diagnosis. Obviously size does matter given the association between concerning size and the pathological diagnosis of well-differentiated liposarcoma [2]. While the association of size to the diagnosis of well-differentiated liposarcoma was not as strong as the variables of stranding and nodularity (Figures 3 and 4), all these represent variables that need to be considered when rendering an ultimate radiographic impression.

Recently, the identification and gene amplification of the MDM2 gene have been identified to supplement the pathological diagnosis of liposarcoma [14, 15]. Unfortunately, the majority of the patients utilized in this study did not have MDM2 FISH for the pathological diagnosis. For the majority of patients in this study, the pathological analysis and diagnosis were made without usage of MDM2 gene amplification. This represents a limitation to this study. For this study, pathological review and diagnosis were made by one of three pathologists with subspeciality training in musculoskeletal oncology and sarcoma pathology following WHO criteria for the classification of sarcoma.

At tertiary referral sarcoma centers, where the treatment team understands the low metastatic potential of these lesions and the appropriateness of a marginal excision, the inability to differentiate these lesions on MRI alone does not represent a major treatment dilemma, as symptomatic tumors require marginal excision without adjuvant treatment [5, 6, 16]. On the contrary, at smaller centers with less experience in musculoskeletal oncology or the specific biology of well-differentiated liposarcoma, statements such as “liposarcoma cannot be excluded,” or “referral to sarcoma center is necessary,” are all too common. Phrases such as these lead to patient worry and unnecessary referral. It has been proposed to characterize all such lesions as “low-grade fatty tumors” which at worst represent well-differentiated liposarcomas. Then, with an understanding of the low metastatic risk and appropriate management of these tumors, treating physicians could more appropriately counsel patients and avoid unnecessary referral.

## 5. Conclusion

Experienced observers in musculoskeletal radiology and orthopaedic oncology can differentiate between lipomas and well-differentiated liposarcomas in 69% of cases. This level of accuracy needs to be improved upon with accepted and validated mechanisms to differentiate between these two entities. The variables of nodularity, stranding, and relative size do show an association with the diagnosis of well-differentiated liposarcoma and therefore should continue to be used in the radiographic impression. A better understanding of the biology of well-differentiated liposarcomas, appropriate treatment, and risk of local recurrence/overall survival related to this diagnosis is important to appropriately counsel patients and avoid unnecessary referral.

## Figures and Tables

**Table 1:** The interrater reliability for diagnosis and each of the categorical variables of stranding, nodularity, and size.

Measure	Kappa estimate	95% CI	Raw proportion of matching responses
Diagnosis	0.63	(0.61, 0.65)	0.82
Stranding	0.17	(0.14, 0.19)	0.76
Nodularity	0.41	(0.39, 0.43)	0.72
Size	0.11	(0.09, 0.13)	0.68

**Table 2:** The relationships of stranding and nodularity to the diagnosis of liposarcoma.

Stranding	Nodularity	% Reader liposarcoma	% True liposarcoma
Yes	Yes	217/228 (95%)	145/228 (64%)
Yes	No	95/268 (35%)	108/268 (40%)
No	Yes	6/11 (55%)	5/11 (45%)
No	No	13/93 (14%)	32/93 (34%)

**Table 3:** The relationship of concerning size to the diagnosis of liposarcoma.

Size	% Reader liposarcoma	% True liposarcoma
Yes	103/138 (75%)	83/138 (60%)
No	228/462 (49%)	207/462 (45%)
